# SNPs Give LACTB Oncogene‐Like Functions and Prompt Tumor Progression via Dual‐Regulating p53

**DOI:** 10.1002/advs.202405907

**Published:** 2024-09-26

**Authors:** Guanyu Huang, Jiajun Zhang, Yu Xu, Fei Wu, Yiwei Fu, Xuelin Zhang, Hanxiao Yin, Yuanyuan You, Peng Zhao, Weihai Liu, Jingnan Shen, Junqiang Yin

**Affiliations:** ^1^ Department of Musculoskeletal Oncology the First Affiliated Hospital of Sun Yat‐sen University Guangzhou 510080 China; ^2^ Guangdong Provincial Key Laboratory of Orthopedics and Traumatology the First Affiliated Hospital of Sun Yat‐Sen University Guangzhou Guangdong China; ^3^ NMPA Key Laboratory for Research and Evaluation of Drug Metabolism & Guangdong Provincial Key Laboratory of New Drug Screening & Guangdong‐Hongkong‐Macao Joint Laboratory for New Drug Screening School of Pharmaceutical Sciences Southern Medical University Guangzhou 510515 China

**Keywords:** clavulanate potassium, LACTB, osteosarcoma, p53

## Abstract

LACTB is identified as a tumor suppressor in several tumors. However, preliminary study reveals that LACTB is overexpressed in osteosarcoma and indicates poor prognosis. Two missense mutations (rs34317102 and rs2729835) exist simultaneously in 92.31% of osteosarcoma patients and cause M5L and R469K double mutations in LACTB, suggesting the biologic function of LACTB protein may be altered in osteosarcoma. Moreover, LACTB^M5L+R469K^ overexpression can promote malignant progression in different tumors, which suggests that the M5L and R469K mutations confer oncogene‐like functions to LACTB. Mechanistically, LACTB^M5L+R469K^ not only reduces the wild type p53 via enhancing PSMB7 catalytic activity, but also protects p53^R156P^ protein from lysosomal degradation, which suggesting LACTB^M5L+R469K^ is a dual‐regulator for wt‐p53 and mutant p53, and derive oncogene‐like functions. More importantly, clavulanate potassium, a bacterial *β*‐lactamase inhibitor, can inhibit osteosarcoma proliferation and sensitize osteosarcoma to cisplatin by binding and blocking LACTB^M5L+R469K^. These findings revealed that the M5L and R469K double mutations can diminish the tumor suppressive ability of wild type LACTB and provide oncogene‐like functions to LACTB. Inhibiting LACTB^M5L+R469K^ can suppress the progression of osteosarcoma harbouring wild‐type or mutant p53. Clavulanate potassium is a promising drug by targeting LACTB^M5L+R469K^‐p53 pathway for the treatment of osteosarcoma patients.

## Graphical abstract

This study shows that the M5L and R469K double mutations diminish the tumor suppressive effect of wild type LACTB and confer oncogene‐like function to LACTB protein. LACTB^M5L+R469K^ is a dual‐regulator for p53 and LACTB^M5L+R469K^ inhibition can both benefit osteosarcoma patients harbouring wt‐p53 or mutant p53. Clavulanate potassium is a potential LACTB^M5L+R469K^ inhibitor and can be used for the treatment of osteosarcoma.

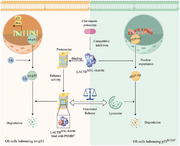

## 1 Introduction

LACTB is a mammalian protein that contains a domain sequence similar to that of penicillin‐binding proteins and *β*‐lactamases.^[^ ^1^, ^2^ ^]^ A recent study showed that LACTB acts as a tumor suppressor in breast cancer and is involved in regulating lipid metabolism.^[^ ^3^ ^]^ After that, more scholars explored the LACTB protein and reported that it played a tumor suppressive role in several tumors, including breast cancer, hepatocellular carcinoma, colorectal cancer and glioblastoma.^[^ ^3^, ^4^, ^5^, ^6^, ^7^, ^8^ ^]^ However, Peng et al. reported that LACTB was highly expressed in the nasopharyngeal carcinoma tissues and promoted metastasis via activation of ERBB3/EGFR‐ERK pathway.^[^ ^9^ ^]^ In our preliminary study, we also identified LACTB as a super enhancer associated gene and unexpectedly revealed that high level of LACTB indicated poor prognosis in osteosarcoma (OS).^[^ ^10^, ^11^ ^]^ These studies suggest that LACTB can function either as an oncogene or as a tumor suppressor, although the mechanisms underlying its opposite roles in tumor are not well understood.

Previous studies have shown that missense mutation can alter protein characteristics; for example, IDH1/2 mutations were identified as early genetic events in brain tumor,^[^ ^12^ ^]^ and KRAS mutations were significantly associated with tumorigenesis and poor survival of patients.^[^ ^13^ ^]^ Single nucleotide polymorphisms (SNPs) have traditionally been considered as harmless mutations. However, some studies have shown that SNPs inducing missense mutation or at special coding regions can increase disease incidence or indicate poor prognosis, such as SNPs of CLPTM1L (rs401681), CIITA (rs6498114) and I148M variant of PNPLA3 (rs738409).^[^ ^14^, ^15^ ^]^ Recent study also showed that the R469K mutation (rs2729835) in LACTB is a naturally occurring, but disease‐causing mutation.^[^ ^16^ ^]^ For example, LACTB with R469K mutation was unable to activate the PISD pathway and lost its tumor suppressive function in MCF7‐RAS and HMLER cancer cells.^[^ ^3^ ^]^ These findings remind us the SNPs of LACTB may alter its functions and need to further study the uncovered role and mechanism of LACTB.

By using whole exome sequencing (WES), we found that 92.31% of OS patients (48/52), 6 OS cell lines (6/6) and two primary OS cells (2/2) carried the M5L and R469K double mutations in LACTB protein simultaneously, suggesting the biologic functions of LACTB may be altered in OS. Furthermore, our study revealed that LACTB^M5L+R469K^ not only promoted malignancy by reducing the protein level of wild‐type p53 (wt‐p53) via enhancing PSMB7 catalytic activity, but also by protecting p53^R156P^ protein from lysosomal degradation. At last, we discovered that clavulanate potassium, a bacterial *β*‐lactamase inhibitor, could inhibit OS proliferation and sensitize OS to cisplatin by binding and blocking LACTB^M5L+R469K^ both in vitro and in vivo.

## 2 Results

### 2.1 LACTB Plays an Oncogenic Role and Indicates Poor Prognosis in Osteosarcoma Patients

**Figure 1:**
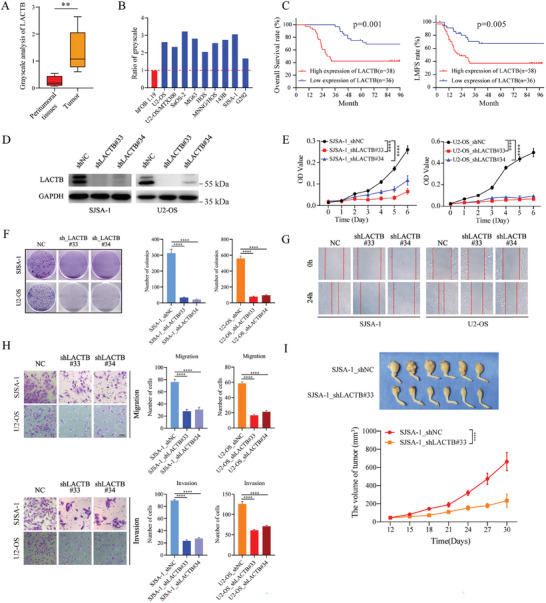
LACTB plays an oncogenic role and indicates poor prognosis in osteosarcoma patients. A) The grayscale analysis of LACTB protein level in OS tissues; paired T test (p = 0.006). B) The LACTB protein level in OS cell lines were compared with normal osteoblast cell line hFOB 1.19 by using western blot and grayscale analysis. C) The overall survival curves and lung metastasis free survival (LMFS) curve were based on the expression level of LACTB in OS tissues from patients who visit our department. D) The LACTB knockdown efficiency were detected by using western blot. E) The proliferation ability of SJSA‐1 and U2‐OS cells in LACTB knockdown group were compared with control group by using CCK‐8. F) Colony formation assays were used to evaluate the colon ability of SJSA‐1 and U2‐OS cells in LACTB knockdown group and control group. G) Scratch assays were used to evaluate the migration ability of SJSA‐1 and U2‐OS cells in LACTB knockdown group and control group. H) Trans‐well assays were used to evaluate the migration and invasion ability of SJSA‐1 and U2‐OS cells in LACTB knockdown group and control group. I) Photographs of resected tumors and tumor growth curves of control group, LACTB knockdown group that using different SJSA‐1 cells injected in proximal tibia.

LACTB has been reported to be a tumor suppressor in breast cancer.^[^ ^3^ ^]^ Contradictory to the results of existing research, our preliminary study identified LACTB as a super enhancer associated gene that promoted tumor progression in OS models.^[^ ^10^, ^11^ ^]^ In the present study, by performing western blot and immunofluorescence, we found that OS tissues had markedly higher LACTB protein expression levels than peritumoral tissues did (**Figure** **1**A; Figure S1A‐C, Supporting Information). We also detected that the level of LACTB protein in OS cell lines was twofold greater than that in the hFOB 1.19 cell line (Figure 1B; Figure S1D, Supporting Information). Further, we found that higher LACTB expression indicated a worse prognosis in OS patients in our department (Figure 1C; Figure S1E, Supporting Information), and consistent results were obtained by screening clinical data from the R2: Genomics Analysis and Visualization Platform (https://r2.amc.nl) (Figure S1F, Supporting Information). These results indicated that LACTB is abnormally high expression in OS and may play a biomarker for the prognosis of OS.

Then, we applied shRNAs to knock down LACTB expression in OS cell lines (Figure 1D; Figure S2A, Supporting Information), and found that LACTB knockdown could reduce the proliferation of U2‐OS, SJSA‐1, and HOS cells (Figure 1E; Figure S2B, Supporting Information). Consistently, the results of the colony formation assay also indicated a significant decrease after LACTB inhibition (Figure 1F; Figure S2C, Supporting Information). The result of scratch experiments and trans‐well assays demonstrated that a low LACTB protein level induced weak migration and invasion in OS cells (Figure 1G,H; Figure S2D, Supporting Information). We established an OS animal model by injecting stable LACTB‐knockdown cells or control cells into the tibia of nude mice. The result showed that tumor volumes were markedly decreased in the LACTB knockdown group compared with those in the control group (Figure 1I). All of these results suggested that LACTB has oncogene‐like functions and promotes progression in OS.

### 2.2 The M5L and R469K Missense Mutations give Oncogene‐Like Functions to the LACTB Protein

**Figure 2:**
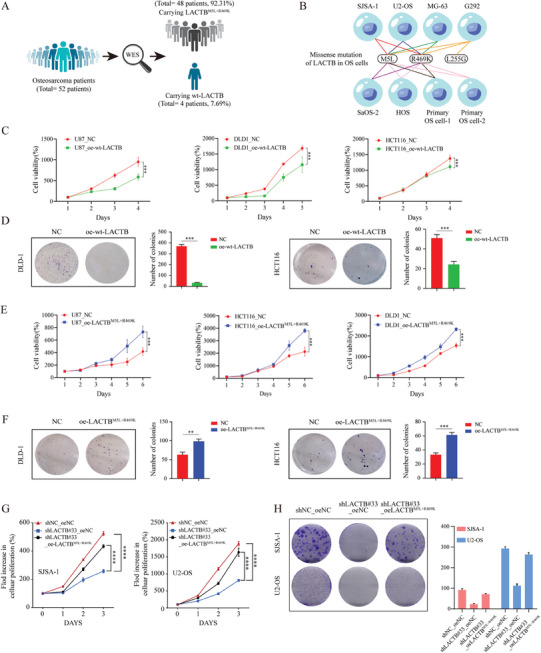
The M5L and R469K missense mutations diminish tumor suppressive function and provide oncogene‐like functions to the LACTB protein. A) Mutation situations of LACTB in 52 OS patient's tissues. B) Mutation situations of LACTB in 6 OS cell lines and 2 OS primary cells. C) The proliferation ability of U87, DLD1, and HCT116 cells in wt‐LACTB over‐expression group were compared with control group by using CCK‐8. D) Colony formation assays were used to evaluate the colony formation ability of DLD1 and HCT116 cells in wt‐LACTB over‐expression group and control group. E) The proliferation ability of U87, DLD1 and HCT116 cells in LACTB^M5L+R469K^ over‐expression group were compared with control group by using CCK‐8. F) Colony formation assays were used to evaluate the colony formation ability of DLD1 and HCT116 cells in LACTB^M5L+R469K^ over‐expression group and control group. G) The proliferation ability of SJSA‐1 and U2‐OS cells in LACTB knockdown group and rescue group were compared with control group by using CCK‐8. H) Colony formation assays were used to evaluate the colon ability of SJSA‐1 and U2‐OS cells in control group, LACTB knockdown group and rescue group.

For investigating the mechanism underlying LACTB promotes OS progression, we detected the LACTB mutation status in OS by using WES. Two missense mutations (rs34317102 and rs2729835) were found to exist simultaneously in 92.31% of the OS patients (48/52), 6 OS cell lines (6/6) and 2 primary OS cells (2/2), which caused M5L and R469K double missense mutations in the LACTB (**Figure** **2**A,B). Then, we induced wild type LACTB (wt‐LACTB)and LACTB^M5L+R469K^ overexpression in different tumor cell lines, which had been reported about LACTB before (Figure S3A,B, Supporting Information). We observed that the overexpression of wt‐LACTB suppressed the proliferation of these cell lines (Figure 2C). Consistent results were obtained from colony formation assays (Figure 2D). Conversely, overexpression of LACTB^M5L+R469K^ promoted proliferation and colony formation ability of these cells (Figure 2E,F). Because SJSA‐1 and U2‐OS cells express high levels of the LACTB protein, we first knocked down LACTB and subsequently overexpressed LACTB^M5L+R469K^ in LACTB knockdown cells (Figure S3C, Supporting Information). We detected that LACTB^M5L+R469K^ over‐expression could rescue the proliferation and colony formation abilities of LACTB knockdown OS cells (Figure 2G,H). it suggests that our shRNA has no significant off‐target effects, and LACTB^M5L+R469K^ can indeed promote the proliferation and colony formation abilities of OS cells. These results mentioned above indicated that the M5L and R469K mutations could diminish the tumor suppressive effect of wt‐LACTB and confer oncogene‐like functions to the LACTB protein.

### 2.3 Knocking Down LACTB^M5L+R469K^ Upregulates the Wild‐Type p53 Protein at the Posttranscriptional Level

**Figure 3:**
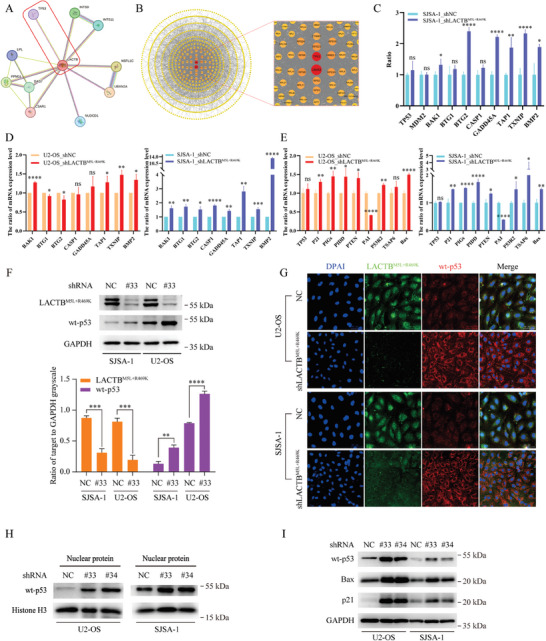
Knocking down LACTB^M5L+R469K^ upregulates the wild‐type p53 protein at the posttranscriptional level. A) The PPI network of LACTB in STRING data base. B) Detecting the core factor of downstream change after LACTB^M5L+R469K^ knockdown by combining RNA‐seq and PPI network analysis. C) The ratio of gene expression between control group and LACTB^M5L+R469K^ knockdown group in RNA‐seq data. D) The RNA‐seq data of LACTB^M5L+R469K^ knockdown were validated by using qPCR. E) Detecting the change of other downstream factors of p53 pathway after LACTB^M5L+R469K^ knockdown by qPCR in U2‐OS and SJSA‐1 cells. F) The wild type p53 protein level of control group and LACTB^M5L+R469K^ knockdown group were checked by western blot and grayscale analysis. G) The wild type p53 protein level of control group and LACTB^M5L+R469K^ knockdown group in U2‐OS and SJSA‐1 cells were detected by cellular immunofluorescence. H) The wild type p53 protein level of nucleus in control group and LACTB^M5L+R469K^ knockdown group were analyzed by western blot. I) The protein level of wild type p53 and downstream factors Bax and p21 in control group and LACTB^M5L+R469K^ knockdown group were checked by western blot.

For studying the specific malignant mechanism of LACTB^M5L+R469K^ in OS cells, we combined the data of STRING database, RNA‐seq and protein–protein interaction (PPI) network analysis revealed that TP53 is the core of the downstream factors after LACTB^M5L+R469K^ knockdown (**Figure** **3**A,B). Because missense mutation in TP53 might invalidate or even reverse its tumor suppressive function, we ensured that U2‐OS and SJSA‐1 cells contained wt‐p53 via the TP53 Database (R20, July 2019: https://tp53.isb‐cgc.org).^[^ ^17^ ^]^ We screened the RNA‐seq data and found that downstream factors of the TP53 pathway, but not the TP53, were markedly altered (Figure 3C), and we validated this result subsequently (Figure 3D). By using qPCR, we also found that the mRNA expression level of TP53 did not change significantly after LACTB^M5L+R469K^ knockdown, but the expression of the TP53 pathway downstream genes changed markedly (Figure 3E). Further, we found that the total and nuclear wt‐p53 protein level were both markedly up‐regulated after LACTB^M5L+R469K^ knockdown (Figure 3F‐H; Figure S4A, Supporting Information). Moreover, two important downstream factors of the TP53 signaling pathway, p21 and Bax, were also markedly up‐regulated in LACTB^M5L+R469K^ knockdown group (Figure 3I; Figure S4B, Supporting Information). These results indicated that LACTB^M5L+R469K^ knockdown could up‐regulate wt‐p53 protein levels through the posttranscriptional approach and stimulate p21 and Bax expression to obtain anti‐tumor effects.

### 2.4 The Regulation of the Wild‐Type p53 Protein by LACTB^M5L+R469K^ is Mediated by PSMB7

**Figure 4:**
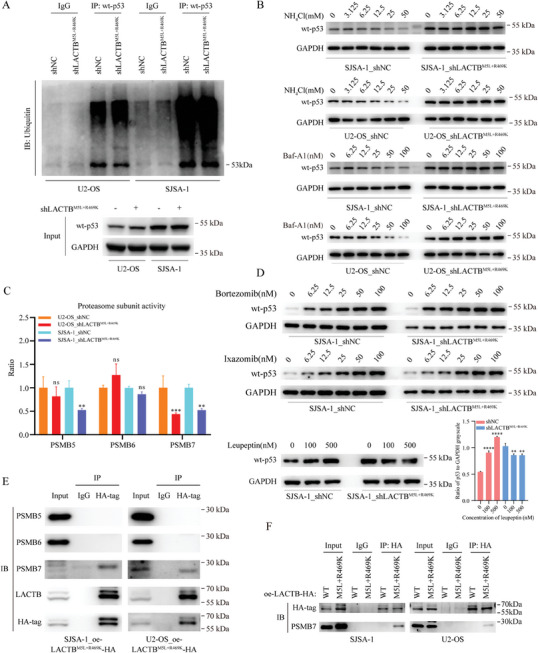
Knocking down LACTB^M5L+R469K^ can upregulate wild type p53 protein via binding and suppressing PSMB7. A) The ubiquitination of wt‐p53 was analyzed by co‐IP and western blot. B) The wild type p53 protein level after treating by lysosome inhibitors for 24 h in control group and LACTB^M5L+R469K^ knockdown group were detected by western blot. C) Proteasome subunits catalytic activity test kit was used to test the catalytic activity of PSMB5, PSMB6, and PSMB7. D) The wild type p53 protein level of SJSA‐1 cells after treating by PSMB5, PSMB6, and PSMB7 inhibitors (Bortezomib/Ixazomib/Leupeptin) for 24 h in control group and LACTB^M5L+R469K^ knockdown group were detected by western blot. E) U2‐OS and SJSA‐1 cells were transfected with indicated plasmid, and then subjected to co‐IP using anti‐HA antibody follow by western blot. F) The wt‐LACTB‐HA and LACTB^M5L+R469K^‐HA overexpression plasmids were transfected into SJSA‐1 and U2‐OS cells, and then subjected to co‐IP using anti‐HA antibody. The protein levels of HA‐tag and PSMB7 were detected by using western blot after co‐IP.

We have found that knocking down LACTB^M5L+R469K^ did not affect the transcriptional action of the TP53, but upregulate wt‐p53 protein at post‐transcriptional level, which suggesting that LACTB^M5L+R469K^ may affect the degradation process of wt‐p53 protein. Because wt‐p53 protein is mainly degraded by the ubiquitin‐proteasome system, and the changing of p53 ubiquitination level or proteasome catalytic activity can affect wt‐p53 degradation. Thus, we first found that knocking down LACTB^M5L+R469K^ had no significant effect on the ubiquitination level of wt‐p53 protein (**Figure** **4**A). We also tested other factors that could affect the level of wt‐p53 protein, but did not obtain positive results (Figure S5A‐C, Supporting Information). By using different dose of Nutlin‐3 (a MDM2 and wt‐p53 interaction disruptor) in control and LACTB^M5L+R469K^ knockdown group, we found Nutlin‐3 had no antagonism effect with LACTB^M5L+R469K^ knockdown (Figure S5D, Supporting Information). It indicated that LACTB^M5L+R469K^ knockdown did not affect the interaction of MDM2 and wt‐p53. All these results suggest that the regulation of the wt‐p53 protein by LACTB^M5L+R469K^ may through downregulating proteasome catalytic activity. Because of the functional balance between the proteasome and lysosome systems, to prove that inhibiting lysosomes can cause proteasome hyperfunction, we used two lysosome inhibitors (NH_4_Cl and Baf‐A1) to induce proteasome hyperfunction. The results showed that wt‐p53 decreased as the drug concentration increased in the control group, but these phenomena disappeared when LACTB^M5L+R469K^ was knocked down (Figure 4B; Figure S5E‐H, Supporting Information). These findings validate that LACTB^M5L+R469K^ inhibition could impair the degradation activity of proteasome. To investigate which proteasome subunit mediates the regulation of LACTB^M5L+R469K^, we applied the proteasome subunit catalytic activity test and found that the PSMB7 subunit catalytic activity decreased dramatically in the LACTB^M5L+R469K^ knockdown group, while the catalytic activity of the PSMB5 and PSMB6 subunits did not change significantly (Figure 4C). Consistently, we used proteasome subunit inhibitors^[^ ^18^ ^]^ (bortezomib, ixazomib and leupeptin) to treat the control group and LACTB^M5L+R469K^ knockdown group, and found that the PSMB7 inhibitor leupeptin could not increase the wt‐p53 protein level of the LACTB^M5L+R469K^ knockdown group cells, while PSMB5 and the PSMB6 inhibitor could still increase the wt‐p53 protein level (Figure 4D; Figure S6A‐I, Supporting Information). Further, we detected that LACTB^M5L+R469K^ could bind to PSMB7 instead of binding to PSMB5 or PSMB6 by co‐immunoprecipitation (co‐IP) (Figure 4E). After we induced wt‐LACTB and LACTB^M5L+R469K^ expression in OS cells, we found wt‐LACTB could not bind PSMB7 protein by using co‐IP. It suggests that M5L and R469K missense mutation are crucial for the binding of LACTB to PSMB7 (Figure 4F). These results suggested that LACTB^M5L+R469K^, instead of wt‐LACTB, could bind PSMB7 subunit and enhance proteasome degradation activity.

To further validate this result, we used siRNAs to knock down PSMB7 (Figure S6J, Supporting Information) and found that, comparing with LACTB^M5L+R469K^ knockdown, PSMB7 knockdown led to similar changes in the expression of TP53 downstream genes (Figure S6K, Supporting Information). Moreover, the western blot results indicated that knocking down PSMB7 could also increase the wt‐p53 protein level markedly (Figure S6L,M, Supporting Information). Here, we reported that LACTB^M5L+R469K^ could bind and up‐regulate PSMB7 catalytic activity and prompting wt‐p53 degradation by proteasome.

### 2.5 Knocking Down LACTB^M5L+R469K^ Promotes p53 ^R156P^ Degradation by Lysosome

**Figure 5:**
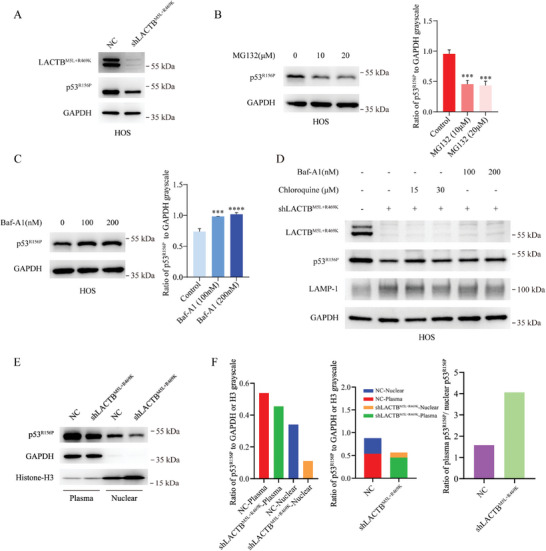
Knocking down LACTB^M5L+R469K^ induced p53 ^R156P^ nuclear exportation and promoted p53 ^R156P^ degradation by enhancing lysosome function. A) HOS cells were transfected with indicated shRNA, and then the protein level of p53^R156P^ in control group and LACTB^M5L+R469K^ knockdown were analyzed by western blot. B) HOS cells were treated with different concentration MG132 for 6 h, and then the p53^R156P^ protein level was analyzed with western blot and grayscale analysis. C) HOS cells were treated with different concentration Baf‐A1 for 6 h, and then the p53^R156P^ protein level was analyzed with western blot and grayscale analysis. D) HOS cells were treated with indicated drugs and shRNA. The protein level of LACTB^M5L+R469K^, p53^R156P^ and LAMP‐1 were detected by western blot. Chloroquine was applied for 24 h both in 15 and 30 µm group. Baf‐A1 was applied for 6 h in 100 nm group and 200 nm group. E,F) The nuclear and cytoplasmic p53^R156P^ protein were extracted, and then analyzed by western blot and grayscale analysis.

In this study, we revealed the mechanism of LACTB^M5L+R469K^ regulating wt‐p53. However, we also observed that LACTB^M5L+R469K^ inhibition could also suppress HOS cells (Figure S2A‐D, Supporting Information), which carry a mutant p53 protein with a p.R156P missense mutation (TP53 Database (R20, July 2019): https://tp53.isb‐cgc.org).^[^ ^17^ ^]^ These findings suggested that LACTB^M5L+R469K^ inhibition could benefit not only the OS patients harbouring wt‐p53 but also those OS patients with p53 mutation. We further found that LACTB^M5L+R469K^ knockdown dramatically decreased the p53^R156P^ protein level in HOS cells (**Figure** **5**A). To investigate whether the proteasome mediates the degradation of p53^R156P^ protein, we first treated HOS cells with the proteasome inhibitor MG132 for 6 h and detected that p53^R156P^ protein level decreased significantly, instead of increasing (Figure 5B). Due to the functional balance between proteasome and lysosome, it suggesting that MG132 could induce lysosome hyperfuction and promote p53^R156P^ protein degradation through lysosome. Then, we treated HOS cells with the lysosome inhibitor Baf‐A1 for 6 h, and observed that the lysosome inhibitor Baf‐A1 could rescue the p53^R156P^ protein level (Figure 5C). It indicates that the lysosome system indeed mediates the degradation of p53^R156P^ protein in HOS cells. We further tested lysosomal functions by detecting the protein level of LAMP‐1, which is a lysosomal membrane protein, and observed that LAMP‐1 was upregulated and the p53^R156P^ protein was downregulated after LACTB^M5L+R469K^ knockdown (Figure 5D). After lysosome inhibitor (Baf‐A1 or chloroquine) treatment, we detected that Baf‐A1 and chloroquine can both rescue p53^R156P^ protein level, and LAMP‐1 was downregulated at the same time (Figure 5D). Due previous studies showed that the mutant p53 protein may form complexes and calculate in the nucleus, which can protect mutant p53 protein from degradation and promote tumor progression.^[^ ^19^, ^20^, ^21^ ^]^ We separated the nuclear and cytoplasmic proteins and found that nuclear p53^R156P^ was decreased and the ratio of cytoplasmic p53^R156P^ to nuclear p53^R156P^ was increased in the LACTB^M5L+R469K^ knockdown group (Figure 5E,F; Figure S7A, Supporting Information). Furtherly, we overexpressed wt‐LACTB‐HA in HOS and HOS_shLACTB^M5L+R469K^ cells, and found wt‐LACTB did not affect p53^R156P^ protein levels significantly in both HOS and HOS_shLACTB^M5L+R469K^ group (Figure S7B, Supporting Information). These results indicated that LACTB^M5L+R469K^ inhibition could not only up‐regulate wt‐p53, but also drive p53^R156P^ nuclear exportation and prompt p53^R156P^ degradation by inducing lysosomal hyperfunction.

### 2.6 Clavulanate Potassium can Suppress Osteosarcoma by Binding and Blocking LACTB^M5L+R469K^

**Figure 6:**
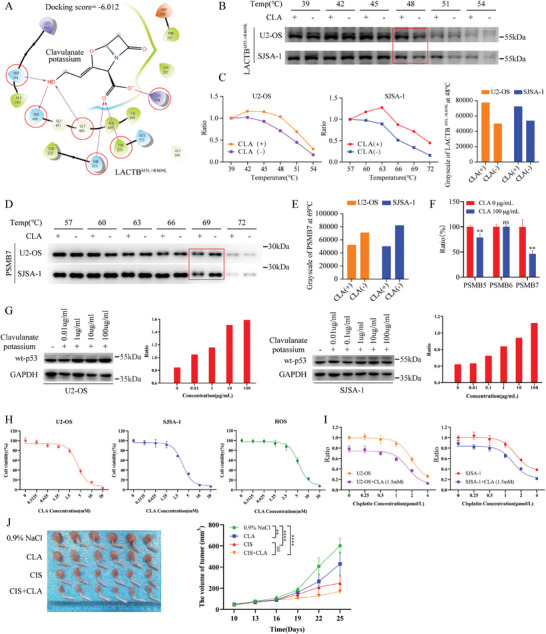
Clavulanate potassium suppresses tumor growth both in vitro and in vivo by inhibiting LACTB^M5L+R469K^. A)The possibility of combination between LACTB^M5L+R469K^ and p53^R156P^ was detected by virtual screen platform. B) Cellular Thermal Shift Assay was used to detected the thermostability of LACTB^M5L+R469K^ in vitro. C) The melting curves of LACTB^M5L+R469K^ protein, and the data were based on the result of western blot. The grayscale of LACTB^M5L+R469K^ 48 centigrade. D) Cellular Thermal Shift Assay was used to detected the thermostability of PSMB7 in vitro. E) The grayscale of PSMB7 protein at 69 centigrade. F) Treating U2‐OS cells with 100ug mL^−1^ CLA for 48 h, then detected the proteasome subunit catalytic activity. G) U2‐OS and SJSA‐1 cells were treated with different concentration CLA for 72 h, and then wild type p53 protein level was analyzed by western blot and grayscale analysis. H) U2‐OS, SJSA‐1 and HOS cells were treated with CLA for 48 h, and the inhibiting efficiency was detected by CCK‐8. I) U2‐OS, SJSA‐1 cells were treated with 1.5 mm CLA and different concentration cisplatin, and then detecting cell viability by CCK‐8. J) Photographs of resected tumors and tumor growth curves of 0.9% NaCl group, CLA group, CIS group and CLA+CIS group that using different SJSA‐1 cells injected in proximal tibia. The symbol of ## means the significant by using T test.

Because the LACTB protein has a similar amino acid sequence to that of bacterial *β*‐lactamase,^[^ ^1^, ^2^ ^]^ it might contain a similar tertiary structure to that of bacterial *β*‐lactamase. So, we investigated the binding possibility between bacterial *β*‐lactamase inhibitor and the LACTB^M5L+R469K^ protein via a virtual screen platform. The result showed that clavulanate potassium (CLA) could bind LACTB^M5L+R469K^ with a pretty low binding energy (docking score = −6.012) (**Figure** **6**A). Due the thermostability of proteins can be altered by binding to a small molecule.^[^ ^22^ ^]^ We further applied Cellular Thermal Shift Assay (CETSA) and found that the thermostability of the LACTB^M5L+R469K^ protein increased after treatment with 1.5 mm CLA (Figure 6B), the melting curves of the LACTB^M5L+R469K^ protein were shifted to the right (Figure 6C), and the most significant difference in the LACTB^M5L+R469K^ protein was at 48 centigrade (Figure 6C). In further, we found the thermostability of the PSMB7 protein decreased significantly after 1.5 mm CLA treatment (Figure 6D), and the most significant differences in the PSMB7 proteins were at 69 centigrade (Figure 6E). We testified these results in endogeneity manner and found the binding of LACTB^M5L+R469K^ and PSMB7 was disrupted partially after 1.5 mm CLA treatment (Figure S8A, Supporting Information). In addition, we treated control and LACTB^M5L+R469K^ knockdown group cells with different concentrations of CLA and detected that CLA did not further increase wt‐p53 protein levels after the LACTB^M5L+R469K^ was knocked down (Figure S8B, Supporting Information). These results indicated that LACTB^M5L+R469K^ protein is a target of CLA, and CLA could disrupt the interaction between LACTB^M5L+R469K^ and PSMB7 protein.

For investigate whether CLA could inhibit the process of LACTB^M5L+R469K^ regulating wt‐p53 via PSMB7, we detected proteasome subunit activity and found that CLA could also decrease PSMB7 catalytic activity significantly after 48 h treatment (Figure 6F). Moreover, CLA could increase the wt‐p53 protein levels of U2‐OS and SJSA‐1 cells in a dose‐dependent manner (Figure 6G). In addition, after treating HOS cells with CLA, we observed that the p53^R156P^ protein level decreased significantly (Figure S8C, Supporting Information), and this result was similar to that observed following LACTB^M5L+R469K^ knockdown. These findings indicated that CLA could up‐regulate wt‐p53 and down‐regulate p53^R156P^ protein level by blocking LACTB^M5L+R469K^.

**Figure 7:**
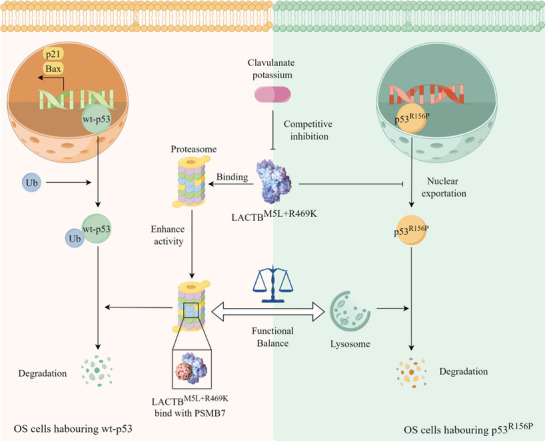
The sketch map of this study.

To detect whether CLA could suppress OS progression via inhibiting LACTB^M5L+R469K^. We treat OS cells with CLA, and found that CLA suppressed U2‐OS, SJSA‐1 and HOS cells at millimolar concentrations, with IC50 of ≈4.12, 3.586, and 5.85 mm, respectively (Figure 6H). We also observed that CLA suppressed the migration of U2‐OS, SJSA‐1 and HOS cells through scratch assay (Figure S9A,B, Supporting Information). Moreover, we determined that CLA and cisplatin (CIS) could have additive effects in vitro (Figure 6I). Then, we established an OS animal model by injecting SJSA‐1 cells into the tibia of nude mice, which were subsequently treated with 0.9% NaCl, CLA, CIS or CIS+CLA. As shown in Figure 6J, the tumor volume in the CIS+CLA group was significantly lower than that in the 0.9% NaCl, CLA and CIS groups. All these results indicated that CLA not only could suppress OS progression in OS‐bearing mouse models by blocking LACTB^M5L+R469K^, but also could enhance the anti‐tumor effect of cisplatin with few side effects. The sketch map of this study is shown in **Figure** **7**.

## 3 Discussion

OS is a highly heterogeneous bone‐derived malignant tumor and lack of effective therapeutic targets. Currently, patients with OS are primarily treated with neoadjuvant chemotherapy and surgical resection. The first‐line chemotherapy drugs include doxorubicin, cisplatin and methotrexate. However, patients with advanced metastatic OS often develop resistance to these chemotherapy drugs, and there are seldom other effective targeted drugs as second‐line treatment options, resulting in extremely poor prognosis for patients with advanced OS, with a 5‐year survival rate of less than 20%. In our preliminarily study, we also identified LACTB as a super enhancer associated gene and revealed that high level of LACTB indicated poor prognosis in OS.^[^ ^10^, ^11^ ^]^ It suggests that LACTB may be a key factor driving the malignant progression of OS, and targeted inhibition of LACTB may be a potential therapeutic strategy.

LACTB is a mammalian protein that contains similar domain to that of bacterial *β*‐lactamases.^[^ ^1^, ^2^ ^]^ Recently, LACTB has been identified as a tumor suppressor first in breast cancer and is involved in lipid metabolism.^[^ ^3^ ^]^ Then, several studies reported that LACTB protein levels were down‐regulated in tumor tissues, and LACTB overexpression could inhibit the proliferation, invasion, and migration of hepatocellular carcinoma, colorectal cancer and glioma via different signaling pathway.^[^ ^4^, ^5^, ^6^, ^7^ ^]^ Another study also showed that low expression of LACTB was associated with a poor response of patients with advanced gastric cancer to neoadjuvant chemotherapy.^[^ ^23^ ^]^ Although studies above mentioned indicate the tumor suppressive role of LACTB, the role of LACTB still has controversy.^[^ ^24^ ^]^ Peng et.al reported that LACTB promoted metastasis of nasopharyngeal carcinoma via activating ERBB3/EGFR‐ERK pathway.^[^ ^9^ ^]^ Xie et.al reported that LACTB was involved in the development of pancreatic adenocarcinoma, and that high LACTB expression level predicted a poor prognosis.^[^ ^25^ ^]^ Our results indicated that LACTB could play an oncogene‐like role in OS and these unexpected findings encourage us to explore LACTB deeply.

R469K mutation of LACTB has been defined as germline mutation and described in SNP database (rs2729835). Although SNPs are commonly deemed as non‐oncogenic factors in most cases, the location of SNP at special coding regions may increase disease incidence or indicate poor prognosis. For example, SNPs of CLPTM1L (rs401681) and CIITA (rs6498114) are located in the regulatory regions of nearby genes and indicate poor prognosis in nasopharyngeal carcinoma patients.^[^ ^14^ ^]^ Besides, missense mutation derive by SNP also could induce disease, such as I148M variant (rs738409) of PNPLA3 was strongly associated with non‐alcoholic fatty liver disease.^[^ ^15^ ^]^ Interestingly, a recent study mentioned that the R469K mutation in LACTB is a naturally occurring, but disease‐causing mutation.^[^ ^16^ ^]^ Furthermore, over‐expression of LACTB^R469K^ did not play any tumor suppressive function in MCF7‐RAS and HMLER cancer cells; on the contrary, wt‐LACTB over‐expression could inhibit the proliferation of MCF7‐RAS and HMLER cancer cells.^[^ ^3^ ^]^ These studies remind us that SNPs should not be ignored and the SNPs of LACTB may have some unknown functions that are worth to be researched deeply.

By using WES, we first found that two SNPs, which cause the M5L and R469K missense mutations, of LACTB exist simultaneously in 92.31% of the OS patients (48/52), 6 OS cell lines (6/6) and 2 primary OS cells (2/2). It suggests that M5L and R469K mutations may alter biologic function of LACTB protein. As we observed, over‐expression of wt‐LACTB suppressed the proliferation of cancer cells. Conversely, overexpression of LACTB^M5L+R469K^ promoted the proliferation and colony formation abilities. The opposite results indicate that the M5L and R469K double mutations may diminish tumor suppressive role of wt‐LACTB and confer oncogene‐like function to LACTB. However, the particular effect of the M5L and R469K mutations is still unclear. A previous study showed that the M5L mutation is located at the N‐terminal mitochondrial localization signal region,^[^ ^2^ ^]^ which suggests the M5L mutation might cause a change in the subcellular localization of the LACTB protein. Additionally, the R469K mutation was reported to be located at one of the three catalytic or substrate‐docking domain of LACTB protein and will take a significant decrease of LACTB catalytic ability.^[^ ^16^ ^]^ Therefore, these two missense mutations might be crucial for the characteristics of LACTB and undoubtedly need to be further explored in the future.

To investigate the mechanism of LACTB^M5L+R469K^ in OS, we used RNA‐seq and PPI network analysis and revealed that TP53 is the downstream factor of LACTB^M5L+R469K^. We found that inhibiting LACTB^M5L+R469K^ could upregulate wt‐p53 by suppressing PSMB7 catalytic activity. More importantly, LACTB^M5L+R469K^ could bind PSMB7 while wt‐LACTB could not. PSMB7 is one of the core catalytic subunits of proteasome. Wu et al. reported that PSMB7 upregulation in multiple myeloma is related to bortezomib resistance and poor prognosis.^[^ ^26^ ^]^ And other studies showed that high PSMB7 expression indicated poor prognosis in patients with colon cancer and breast cancer.^[^ ^27^, ^28^ ^]^ Our study first reports that M5L and R469K mutations are crucial for the binding of LACTB to PSMB7, and PSMB7 mediates the regulation of LACTB^M5L+R469K^ to wt‐p53 protein, and targeting PSMB7 could be valuable strategy for regulating wt‐p53 to treat tumor.

Moreover, our study also shows that inhibiting LACTB^M5L+R469K^ could drive p53^R156P^ nuclear export and promote p53^R156P^ degradation by enhancing lysosomal activity in HOS cells. The mutation of TP53 may abrogate the tumor suppressive ability of p53 or even lead to gain of function (GOF).^[^ ^29^, ^30^, ^31^ ^]^ Several studies have shown that the mutant p53 protein is difficult to be degraded via the ubiquitin‐proteasome system and may form complexes in the nucleus to promote tumor progression.^[^ ^19^, ^20^, ^21^ ^]^ Alexandrova et al. reported that HSP90 can attenuate the degradation of mutant p53, and HSP90 inhibition can prolong survival in a conditional inactivatable p53^R248Q^ (floxQ) mice model by promoting mutant p53 degradation.^[^ ^32^ ^]^ Another study reported that the MCB613 is a promising inhibitor, which suppressed ovarian cancer cells by driving p53^R175H^ nuclear export and promoting p53^R175H^ degradation by lysosome.^[^ ^21^ ^]^ Our results indicate that inhibiting LACTB^M5L+R469K^ has similar effects compared with MCB613 and suggest that LACTB^M5L+R469K^ inhibition is a reliable means of depleting mutant p53^R156P^ protein. And the detailed mechanism under which LACTB^M5L+R469K^ regulates the lysosomal degradation of p53^R156P^ still needs to be explored.

The above results show that LACTB^M5L+R469K^ not only reduces the protein level of wt‐p53 via enhancing PSMB7 catalytic activity in OS, but also protects p53^R156P^ protein from lysosomal degradation in tumors harbouring p53^R156P^ mutation, which suggests that LACTB^M5L+R469K^ could be a dual‐regulator for regulating p53 protein in tumor and inhibiting LACTB^M5L+R469K^ is an attractive strategy for the treatment of OS, which can take advantage to patient harbouring wt‐p53 or p53^R156P^ mutation.

Recently, anti‐tumor drugs that targeted p53, such as nutlin‐3, RG7112 and idasanutlin, usually cause some nonnegligible complications due to their blocking effect is too extensive, which prevents their further clinical translation.^[^ ^33^, ^34^, ^35^, ^36^, ^37^ ^]^ Other MDM2‐p53 PPI disruptors, such as APG‐115 and AMG232, also cause similar severe side effects.^[^ ^38^, ^39^, ^40^, ^41^, ^42^ ^]^ Therefore, it is an urgent requirement to develop novel strategies for restoring the tumor suppressive function of p53. Inspired by the similar tertiary structure between LACTB and bacterial *β*‐lactamase,^[^ ^1^, ^2^ ^]^ we found that clavulanate potassium (CLA), a bacterial *β*‐lactamase inhibitor commonly used in clinical antibiotic therapy, was able to bind to LACTB^M5L+R469K^ through molecular docking and CETSA. The results of co‐IP also indicated that CLA could disrupt the binding of LACTB^M5L+R469K^ to PSMB7. Consistent with the effect of LACTB^M5L+R469K^ knockdown, CLA can both upregulate wt‐p53 protein levels and deplete p53^R156P^, which suggests CLA is a potential inhibitor of LACTB^M5L+R469K^ and could suppress OS progression. Notably, our study showed that CLA could suppress the proliferation and migration abilities of OS cells and that it could sensitize OS cells to cisplatin both in vitro and in vivo. More excitingly, CLA has been widely used in clinic and its bio‐safety has been confirmed by earlier studies.^[^ ^43^, ^44^, ^45^, ^46^ ^]^ It indicates that comparing with other anti‐tumor drugs, CLA has fewer side effects and easier for clinical transformation. On the basis of our study, we first reported that CLA could suppress OS progression by blocking LACTB^M5L+R469K^ and propose that CLA is a promising anti‐tumor agent with less side effects for anti‐tumor via targeting LACTB^M5L+R469K^‐p53 pathway.

In general, our findings first show that the M5L and R469K double mutations diminish the tumor suppressive effect of wt‐LACTB and confer oncogene‐like function to LACTB protein. LACTB^M5L+R469K^ is a dual‐regulator for p53 protein, and LACTB^M5L+R469K^ inhibition could both benefit OS patients harbouring wt‐p53 or mutant p53. Notably, we discover that CLA is a potential LACTB^M5L+R469K^ inhibitor and can be used for the treatment of OS.

## 4 Experimental Section

### Human Cell Lines and Samples

Human OS cell lines, including U2‐OS, SaOS‐2, MG63, HOS, MNNG/HOS, 143B, SJSA‐1, and G292, were purchased from ATCC. U2‐OS/MTX300 cells, a methotrexate‐resistant derivative of the U2‐OS human OS cell line, were kindly provided by Dr. M. Serra (Instituti Ortopedici Rizzoli, Bologna, Italy). The HEK‐293T, HCT116, U87, and DLD1 cell lines and the human osteoblast cell line hFOB 1.19 were obtained from ATCC. All culture methods for these cells were performed according to the ATCC cell culture instructions.

All patients signed an informed consent form. The study was approved by the Ethics Committee of The First Affiliated Hospital of Sun Yat‐Sen University [2021] 755.

### Cell Culture

The human OS cell lines, HCT116, U87, and HEK‐293T were grown in 1x DMEM (Gibco, Thermo Fisher Scientific) supplemented with 10% FBS (Gibco, Thermo Fisher Scientific) and 1% penicillin/streptomycin (Gibco, Thermo Fisher Scientific). DLD1 cells were grown in RPMI 1640 medium (Gibco, Thermo Fisher Scientific) supplemented with 10% FBS. All tumor cell lines and HEK‐293T cells were incubated in a 37 °C incubator with 5% CO_2_. The human osteoblast cell line (hFOB1.19) was grown in 1:1 F12/DMEM (Gibco, Thermo Fisher Scientific) supplemented with 10% FBS (Gibco, Thermo Fisher Scientific) and 1% penicillin/streptomycin (Gibco, Thermo Fisher Scientific). The hFOB1.19 cells were incubated in a 34 °C incubator with 5% CO_2_.

### Compounds and Antibodies

MG132 (S2619), G418 (S3028), Baf‐A1 (S1413), Chloroquine (S6999), Bortezomib (S1013), Ixazomib (S2180) and Leupeptin (S7380) were obtained from Selleck Corporation. NH_4_Cl (A116373) was purchased from Aladdin. Puromycin was purchased from Sigma. Clavulanate potassium was purchased from Solarbio. The ProteinA/G magnetic beads (L‐1004) was purchased from Biolinkedin. The Proteasome Activity Assay (ab107921) was purchased from Abcam. Antibodies against LACTB (18195‐1‐AP), p53 (60283‐2‐Ig, 10442‐1‐AP), p21 (10355‐1‐AP), Bax (50599‐2‐Ig), MDMX (17914‐1‐AP), VINCULIN (26520‐1‐AP), *β*‐actin (66009‐1‐Ig) and LAMP1 (21997‐1‐AP) were purchased from Proteintech. Antibodies against p53 (#ab26), MDM2 p‐S186 (ab22710), MDM2 p‐S166 (ab170880) and MDM2 (ab16895) were purchased from Abcam. Antibodies against GAPDH (#5174S), histone H3 (#4499S), HA‐tag (#3724S), PSMB5 (#12919S), PSMB6 (#13267S), PSMB7 (#13207S), Ubiquitin (#43124S) and p53 p‐S15 (9284S) were purchased from Cell Signalling Technology. Secondary antibodies, including goat anti‐rabbit IgG H&L (#ab6721), goat anti‐mouse IgG H&L (#ab6789), Goat Anti‐Mouse IgG H&L (Alexa Fluor 488) (ab150113), Goat Anti‐Mouse IgG H&L (Alexa Fluor 647) (ab150115) were purchased from Abcam. HRP‐mouse anti‐rabbit IgG heavy chain specific antibody (SA00001‐7H) and HRP‐mouse anti‐rabbit IgG light chain specific antibody (SA00001‐7 L) were purchased from Proteintech.

### Plasmid Construction and Lentivirus Packaging

Four shRNAs targeting the LACTB gene and matching scramble shRNAs were cloned and inserted into the psi‐LVRU6GP vector. The 3xHA‐tagged LACTB and the matching scramble construct were cloned and inserted into the pReceiver‐Lv157 vector. All these plasmids and vectors were purchased from Genecopoeia. Lenti‐Pac HIV Expression Packaging kits (HPK‐LvTR‐40) were purchased from Genecopoeia, and the packaging steps were performed according to the manufacturer's instructions.

### Cell Transfection

The OS cell line was transfected with recombinant lentiviruses packaged with shRNA or overexpression plasmid, and 10 µg mL^−1^ polybrene was added to the media. Puromycin (1 µg mL^−1^) or G418 (500 µg mL^−1^) was used to select cells at 48 h after transfection. The selection step was stopped when puromycin/G418 was used to eliminate all cells in the nontransfected control group, after which puromycin/G418 was used at a constant concentration in routine culture. The selected concentration and maintenance concentration of puromycin were 1 and 0.5 µg mL^−1^, respectively. The selected concentration and maintenance concentration of G418 were 500 and 200 µg mL^−1^, respectively.

### In Vitro Migration and Invasion Assays

Cell migration and invasion assays were performed using Transwell cell culture chambers with 8‐mm microporous filters. For invasion experiments, the cells were precoated with Matrigel according to the manufacturer's instructions. For the experiments, 200 µL of OS cell suspension (10^4^–10^5^ cells mL^−1^, according to the type of experiment and cell line) in serum‐free DMEM was seeded in the upper chambers of 24‐well plates, and 500 µL of DMEM containing 10% FBS was added to the bottom chamber. The migration and invasion processes were stopped at 12–24 h after seeding. Methanol was used to fix the cells, and crystal violet was used for staining. The number of cells in six random fields was counted under a microscope.

### CCK‐8 Kit

A CCK‐8 kit was used to measure cell viability. The control group and shRNA group of U2‐OS and SJSA‐1 cells were seeded in a 96‐well microplate at a density of 1000 cells per well. These cells were grown in DMEM supplemented with 10% FBS and 1% penicillin/streptomycin. The cells were treated with 10 µL of CCK‐8 reagent and 100 µL of DMEM supplemented with 10% FBS per well. After incubating for 2 h in a 37 °C incubator, the optical density was detected at 450 nm once per day for 7 days. The results were presented as the mean ± SD of three independent experiments.

### Colony Formation Assay

U2‐OS and SJSA‐1 cells (including those in the control group and shRNA group) were seeded in six‐well plates at a density of 1000 cells per well in 2 mL of DMEM containing 10% FBS. Colonies containing >50 cells were counted after 7–10 days by staining with crystal violet. The results were presented as the mean ± SD of three independent experiments.

### RNA Extraction and qPCR

Total RNA was extracted from OS cells and purified by using a Total RNA Kit Ι (OMEGA) according to the manufacturer's instructions. After RNA extraction, reverse transcription was performed using a PrimeScript RT reagent Kit with gDNA Eraser (Takara). qPCR was performed using TB Green Premix Ex Taq ΙΙ (Takara) on a real‐time PCR system. All reactions were carried out in a 20 µL reaction volume and performed in triplicate. The qPCR protocol was performed according to the manufacturer's instructions. All sequences of primers used were obtained from the PrimerBank database (https://pga.mgh.harvard.edu/primerbank/). The relative amount of the target gene mRNA was normalized to that of GAPDH or *β*‐actin. The primers used to amplify the indicated genes are shown in the Table S1 (Supporting Information).

### Western Blot

Proteins were extracted from OS cells using RIPA buffer (Keygen) or cell lysis buffer for IP (Beyotime) with protease and phosphatase inhibitors (Roche) and centrifuged for 20 min at 14,000 rpm and 4 °C. After quantifying the protein concentration by using a BCA kit, ≈10–20 µg of protein lysate was loaded on the gels. The longitudinal electrophoresis protocol involved using 80 V for 30 min and then raising the voltage to 120 V until electrophoresis was complete. After horizontal electrophoresis, the proteins were transferred to polyvinylidene difluoride membranes (Millipore) at 100 V for 70 min. The membranes were blocked with 5% BSA and TBST for 90 min at room temperature. Then, the membranes were incubated with primary antibodies overnight at 4 °C. The membranes were washed three times with TBST on a horizontal shaker and incubated with HRP‐conjugated secondary antibodies for 60 min at room temperature. Chemiluminescent detection was performed with an enhanced chemiluminescence (ECL) system. Densitometry quantification was performed using ImageJ.

### Mouse Xenograft

Animal experiments were approved by the Institutional Review Board of The First Affiliated Hospital of Sun Yat‐sen University and were performed according to established standards for the Use and Care of Laboratory Animals [2020] 393. Four to six week old female nude mice were purchased from the Animal Center of The First Affiliated Hospital of Sun Yat‐sen University. After anaesthetizing, wild‐type or plasmid‐transfected SJSA‐1 cells were injected into the proximal tibia. For each mouse, 20 µL of cell suspension (containing 10^6^ cells) was injected.

For the study of LACTB functions, the mice were randomly divided into three groups. After injection, the mice were carefully nurtured for ≈4 to 5 weeks, after which the tumor size was measured in two perpendicular dimensions (L and W). The tumor volume was calculated using the Formula V = π/6×L×W^2^.

For the study of clavulanate potassium, the mice were randomly divided into four groups. After ≈10–14 days, when the tumor volume reached ≈200 mm^3^, the different drugs were used. These four groups of mice were treated with 0.9% NaCl, 200 mg kg^−1^ clavulanate potassium, 5 mg kg^−1^ cisplatin or 200 mg kg^−1^ clavulanate potassium plus 5 mg kg^−1^ cisplatin every three days. Then, the mice were killed, and the tumors were harvested.

### Nuclear Protein and Cytoplasmic Protein Extraction

The different groups of cells were collected into 1.5 mL EP tubes. Then, following the instructions of the Nuclear and Cytoplasmic Protein Extraction Kit (Keygen), 450 µL of Buffer A and 50 µL of Buffer B were mixed and added to the cell precipitations. The tubes were incubated on ice for 30 min and then centrifuged at 3000 rpm at 4 °C for 10 min. The supernatant was collected into a new centrifuge tube containing the cytoplasmic protein. Then, 100 µL of Buffer C was added to the cell precipitate, which was allowed to settle on ice for 60 min. After that, the cell precipitations were centrifuged at 1400 rpm at 4 °C for 30 min. The supernatant was collected into a new centrifuge tube containing the nuclear protein.

### Clinical Data Analysis and IHC

The clinical data of 74 OS patients were analyzed who had visited our department between 2004 and 2018. The histologic type was confirmed prior to our experiments by pathologists from the Clinical Pathology Department of the hospital. This retrospective analysis of anonymous data was approved by the Institutional Review Board of The First Affiliated Hospital of Sun Yat‐sen University and conducted in accordance with the Declaration of Helsinki.

### Immunofluorescence

The cells were placed in 12‐well plates with slides. The cells were gently washed with PBS, and 75% ethanol was subsequently added to fix the cells. Then, 0.3% Triton solution was added for membrane disruption treatment. After the liquid was removed, the cells were gently washed once with PBS. After blocking with 10% BSA, the primary antibodies (dilution ratio: LACTB 1:150, p53 1:100) were added, and the samples were incubated overnight at 4 °C. The goat anti‐rabbit IgG H&L (Alexa Fluor 488) secondary antibody and goat anti‐mouse IgG H&L (Alexa Flour 647) secondary antibody were applied. After incubation, the sections were incubated with DAPI at room temperature for 10 min. Images were taken using a fluorescence microscope.

### PPI Network Analysis

The RNA‐seq data was analyzed with these standards. ① Remove genes with expression levels<1. ② Genes with a Log2FC ≥ 1.5 were extracted from the SJSA‐1 and U2‐OS cohorts, after which the intersection was determined. After screening the data, the data was analyzed in the STRING database.

### Proteasome Subunits Catalytic Activity Test Kit

Fluorescent substrate configuration: According to the instructions, dissolve the fluorescent substrate LLVY‐R110 (PSMB5 sub‐substrate), Z‐LG‐AMC (PSMB6 sub‐substrate) and in DMSO to prepare a storage solution of 100mm. ^[^ ^18^ ^]^ Use 67.7 µL to dissolve the fluorescent substrate Ac‐ALA‐AMC (PSMB7 subunit substrate)^[^ ^18^ ^]^ in a 5% acetic acid solution to prepare a storage solution of 100 mm. Seed different group cells into a black 96‐well plate. Dissolving 2 µL storage solution into 2 mL assay buffer, and adding 100 µL to each well. After 4 h incubation, the corresponding excitation wavelength and emission wavelength were selected at the multifunctional microplate reader to detect the fluorescence intensity in the wells. LLVY‐R110 λ_Ex_ = 490 nm, λ_Em_ = 525 nm; Z‐LLG‐AMC and Ac‐ALA‐AMC λ_Ex_ = 360 nm, λ_Em_ = 465 nm. The fluorescence intensity represents the activity strength of the corresponding subunit.

### Molecular Docking

Ligand preparation: using Chemdraw software to create the ligand structure as the original 2D file, and import the ligand structure file into Schr ö dinger Maestro 11.5 software. Ligands for molecular docking studies were prepared using LigPrep modules. Under the target pH of Epik ionizer at 7.0 ± 2.0, each ligand produced up to 32 stereochemical structures with appropriate protonation states. The optimization of small molecule structure adopts OPLS3 force field to minimize the energy of small molecules, construct tautomeric and desalted ligands, and generate optimized 3D conformation.

Protein structure pretreatment: the Protein Preparation Wizard module of Schr ö dinger Maestro 11.5 was used to preprocess and detect the structure of proteins, allocating disulfide bonds, bond sequences, and formal charges to obtain the correct shape. Auxiliary factors, metal ions, water molecules with crystal formation distances exceeding 5.00 Å, and heteroatoms were all be eliminated. The H‐bond assignment tool was used to optimize hydrogen bond networks. Use the Receptor Grid Generation module to define the binding sites of protein structures, set the properties to 1.0 van der Waals scale factor and 0.25 charge cutoff, and generate a Grid binding pocket. This way, a series of ligand positions may bind to the expected active sites in the future.

Docking method: in the docking module Ligand Docking, choose to load the zip file of the binding pocket, and then select the optimized ligand that needs to be docked earlier for docking. The maximum number of ligand postures should be set within 20, and the van der Waals scale of the ligand should be 0.80. By using the Ligand Interaction module, protein small molecule interaction maps can be obtained. Molecular docking studies were conducted according to the standard procedure recommended by Schr ö dinger.

### Cellular Thermal Shift Assay

SJSA‐1 and U2‐OS cells were collected by using trypsin and centrifuging at 1500 rpm for 3 min, after which the supernatant was discarded, and 2 mL of PBS was added to resuspend the cells. PBS, the protease inhibitor cocktail and the phosphatase inhibitor were added to each cell pellet, and liquid nitrogen was used to thaw and thaw the cell pellet three times. Then, the samples were centrifuged at 4 °C and rcf = 20 000 g for 20 min, after which the supernatant was collected. After the samples were divided into two equal groups, 1.5 mm clavulanate potassium was added to each group for 30 min, after which the samples were separated into 50 µL per EP tube. Setting the temperature gradient, every sample was heated for 3 min, after which the samples were centrifuged at 4 °C and rcf = 20 000 × g for 20 min, after which the supernatant was collected. Fivefold loading buffer was added to every sample, which was subsequently heated at 100 °C for 10 min. Finally, western blotting was used to detect changes in the melting temperature.

### Statistical Analysis

All statistical analyses were performed by using GraphPad Prism 8.0.1 software or SPSS version 20.0 software. Student's t test or ANOVA was used to compare two groups or more than two groups, respectively. A value of p < 0.05 was considered to indicate a statistically significant difference.

## Conflict of Interest

The authors declare no conflict of interest.

## Author Contributions

G.H., J.Z., Y.X., and F.W. contributed equally to this work. G.H., J.Z., and W.L. performed molecular experiments. Y.X. and F.W. analyzed RNA‐seq and WES data. X.Z. and H.Y. performed animal experiments. P.Z. and Y.Y. performed the virtual screen platform and molecular docking experiment. J.S. and J.Y. supplied ideas and collected clinical samples. G.H., W.L., and J.Y. wrote the manuscript. All co‐authors have seen and approved the manuscript.

## Supporting information



Supporting Information

## Data Availability

The data that support the findings of this study are available from the corresponding author upon reasonable request.
