# Supplementary material for: SNPs Give LACTB Oncogene‐Like Functions and Prompt Tumor Progression via Dual‐Regulating p53
Source: Adv Sci (Weinh). 2024 Sep 26;11(43):2405907. doi: 10.1002/advs.202405907 (PMC11578386; doi:10.1002/advs.202405907)
Supplement: Supplementary file 1 — Supporting Information [file ADVS-11-2405907-s001.docx]

**Supplemental Material**


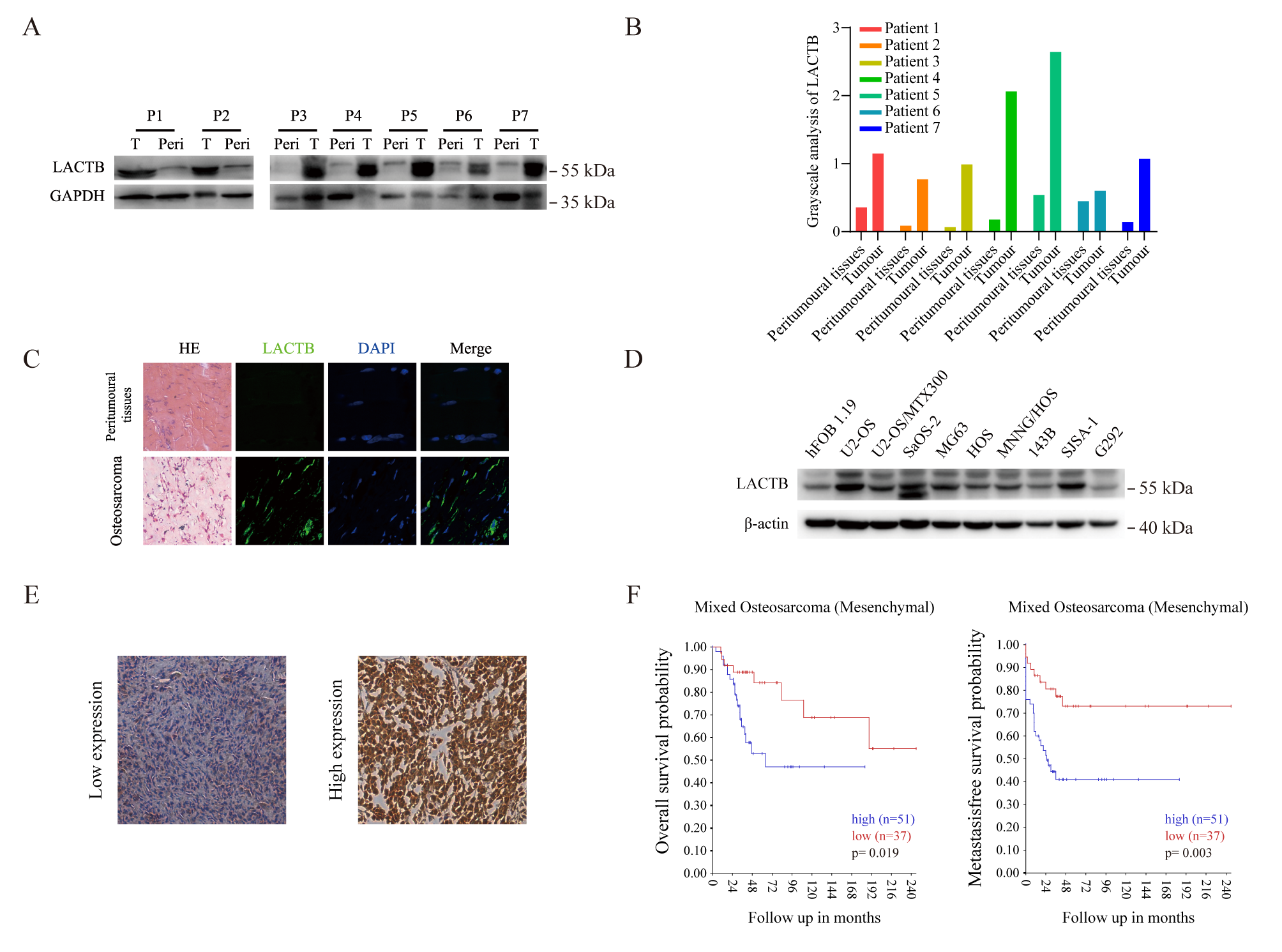


**Figure S1. LACTB highly expressed in osteosarcoma and indicated poor prognosis.** (A)(B) The expression level of LACTB protein in OS tissues and peritumoural tissues were detected by western blot and grayscale analysis. (C) The LACTB protein level of OS tissues were compared with peritumoural tissues by using immunofluorescence. (D) The LACTB protein level in OS cell lines were compared with normal osteoblast cell line hFOB 1.19 by using western blot. (E) The IHC of LACTB in OS tissues from patients who visit our department. (F) The relationship between the expression level of LACTB and overall survival curves or lung metastasis free survival curve of OS patients were obtain from R2 database.

**
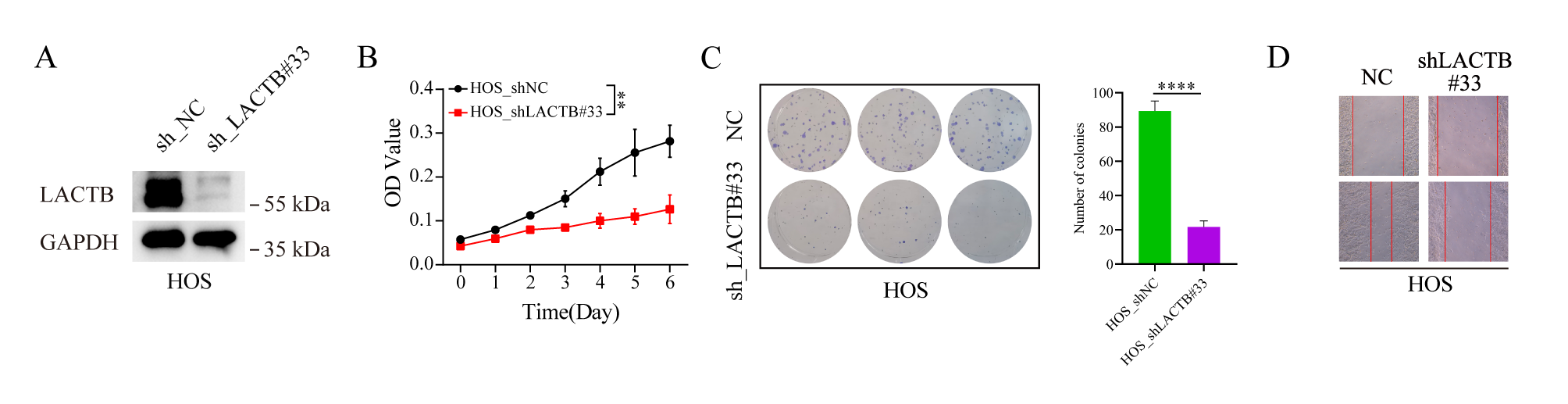
**

**Figure S2. Knocking down LACTB can inhibit malignant ability of HOS cells.** (A) The LACTB knockdown efficiency were detected by using western blot. (B) The proliferation ability of HOS cells in LACTB knockdown group were compared with control group by using CCK-8. (C) Colony formation assays were used to evaluate the colon ability of HOS cells in LACTB knockdown group and control group. (D) Scratch assays were used to evaluate the migration ability of HOS cells in LACTB knockdown group and control group.

**
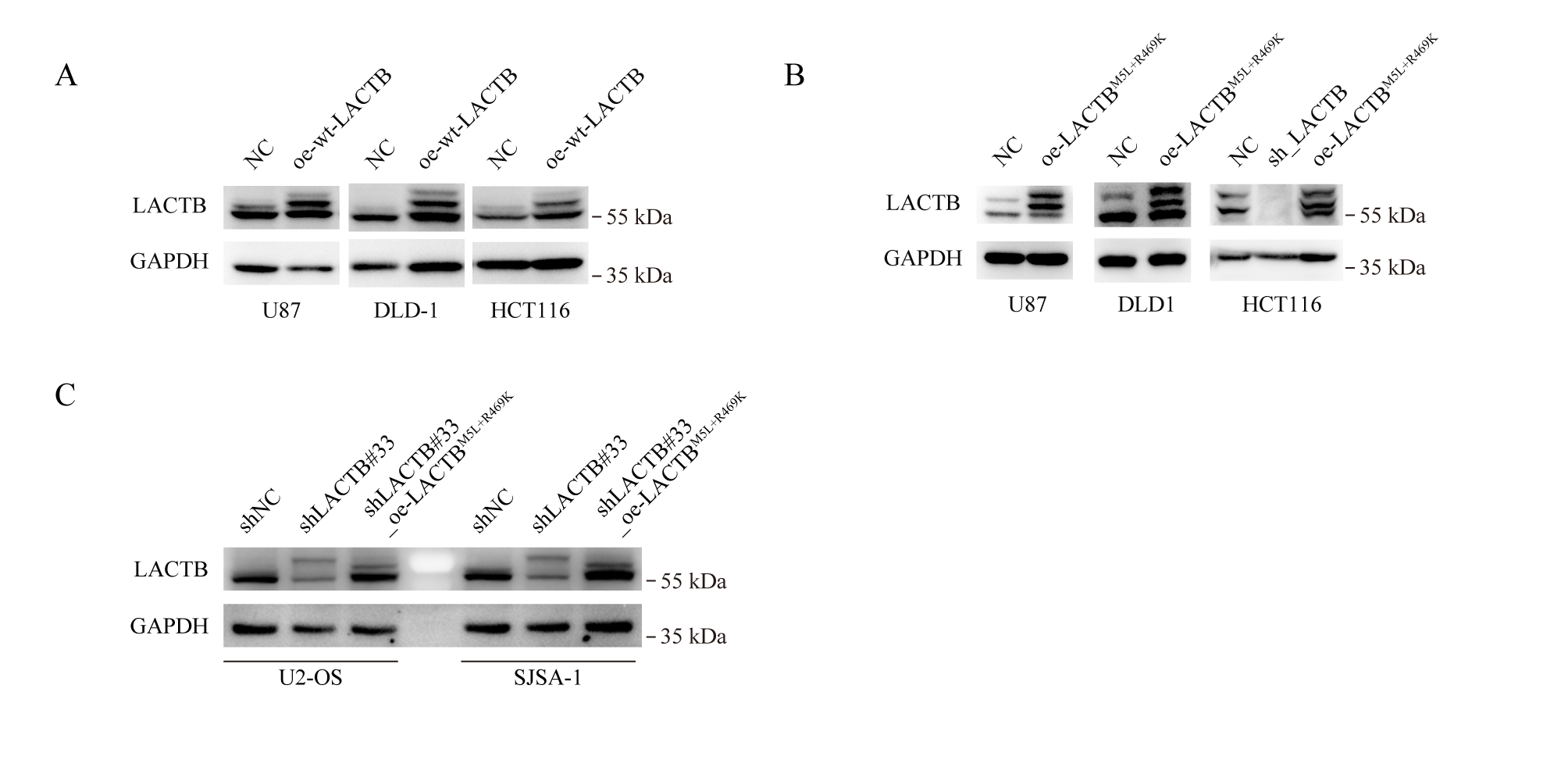
**

**Figure S3. Over-expressed LACTB^M5L+R469K^ and wt-LACTB in different tumour cells.** (A) The efficiency of overexpression of wt-LACTB in U87, DLD1 and HCT116 cells. (B) The efficiency of overexpression of LACTB^M5L+R469K^ in U87, DLD1 and HCT116 cells. (C) The LACTB knockdown and LACTB^M5L+R469K^ rescue efficiency were detected by using western blot.


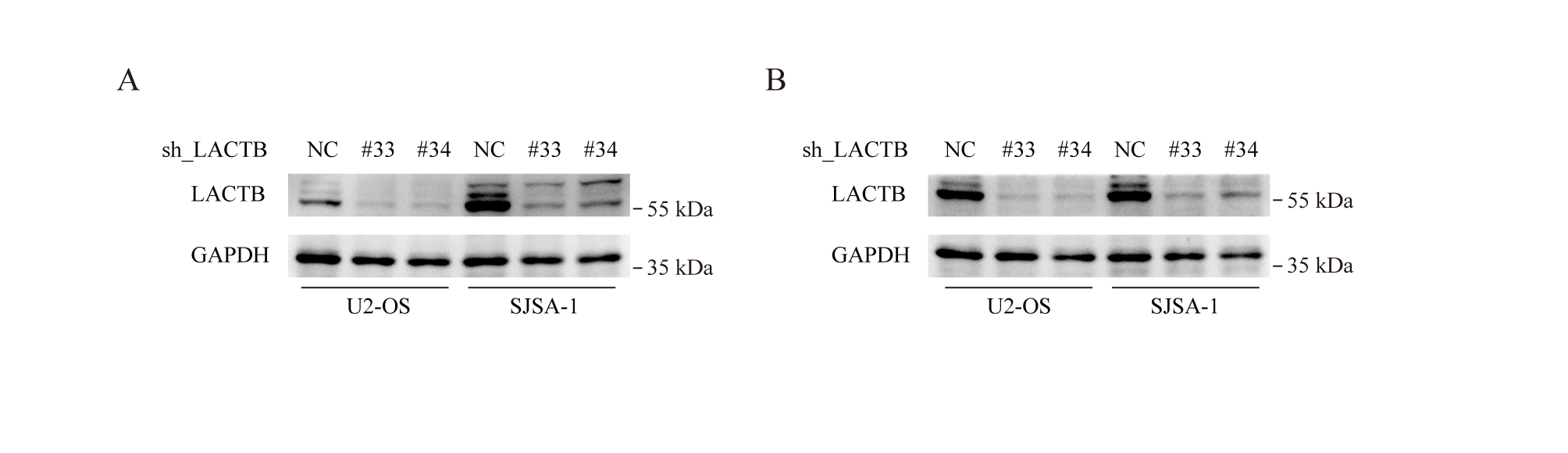


**Figure S4. Detecting the knockdown efficiency of LACTB^M5L+R469K^ in U2-OS and SJSA-1 cells.** (A) (B)The protein levels of LACTB^M5L+R469K^ knockdown were tested by western blot.


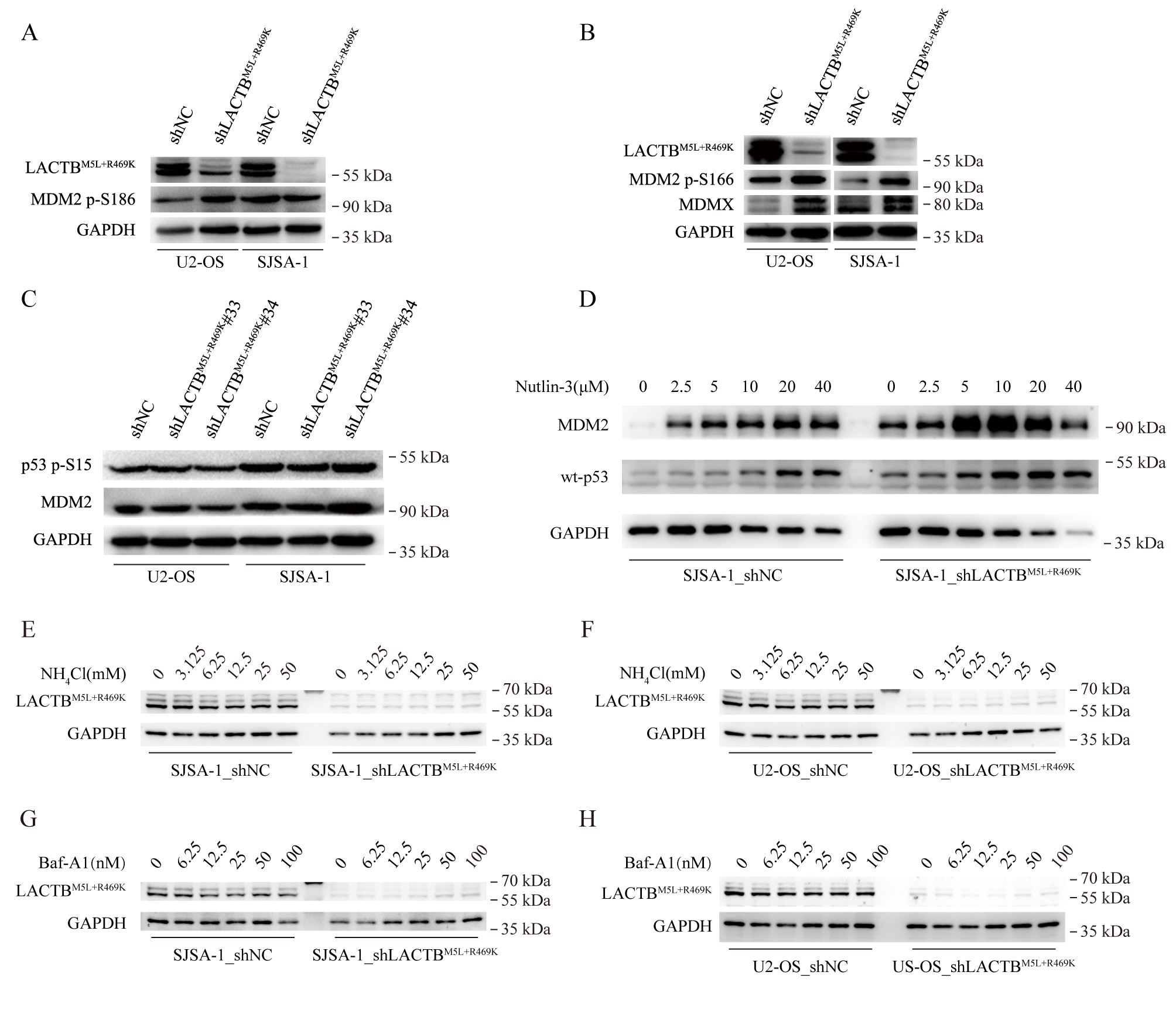


**Figure S5. Detecting other regulation factors of wt-p53 by using western blot.** (A) The protein levels of MDM2 p-S186 after LACTB^M5L+R469K^ knockdown were tested by western blot. (B) The protein levels of MDM2 p-S166 and MDMX after LACTB^M5L+R469K^ knockdown were tested by using western blot. (C) The protein levels of MDM2, p53 p-S15 after LACTB^M5L+R469K^ knockdown were tested by western blot. (D) The protein levels of MDM2 and wt-p53 after nutlin-3 (a MDM2-p53 interaction disruptor) treatment were detected by western blot. The wt-p53 level further increasing in LACTB knockdown group, and showed that LACTB knockdown did not affect the interaction between MDM2 and wt-p53 protein. (E) The LACTB^M5L+R469K^ protein levels after NH_4_Cl treatment for 24h in SJSA-1 control group and LACTB^M5L+R469K^ knockdown group were detected by using western blot. (F) The LACTB^M5L+R469K^ protein levels after NH_4_Cl treatment for 24h in U2-OS control group and LACTB^M5L+R469K^ knockdown group were detected by using western blot. (G) The LACTB^M5L+R469K^ protein levels after Baf-A1 treatment for 24h in SJSA-1 control group and LACTB^M5L+R469K^ knockdown group were detected by using western blot. (H) The LACTB^M5L+R469K^ protein levels after Baf-A1 treatment for 24h in U2-OS control group and LACTB^M5L+R469K^ knockdown group were detected by using western blot.

**
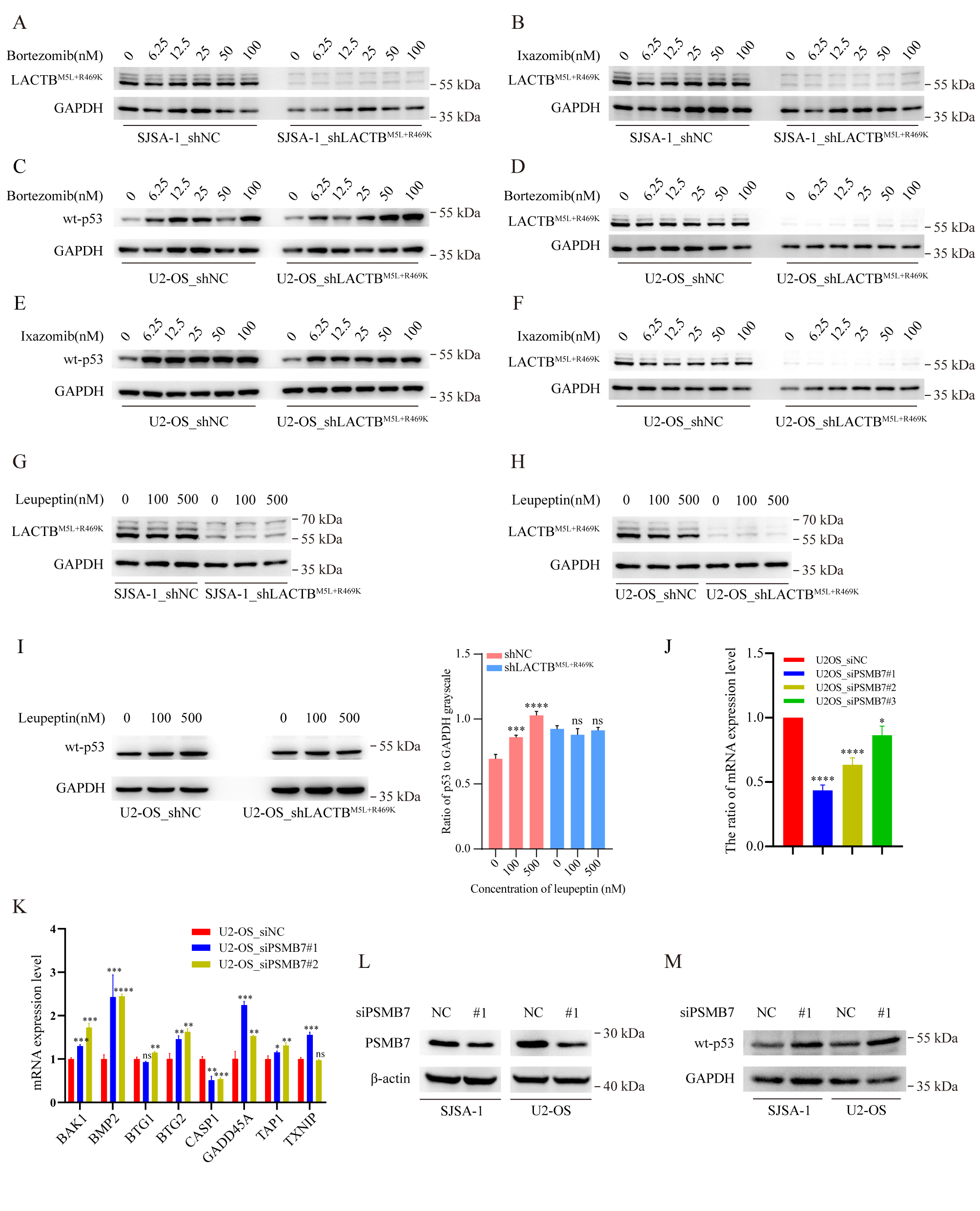
**

**Figure S6. Proteasome subunit PSMB7 mediated the regulation of LACTB^M5L+R469K^ to wt-p53.** (A) The LACTB^M5L+R469K^ protein levels after Bortezomib treatment for 24h in SJSA-1 control group and LACTB^M5L+R469K^ knockdown group were detected by using western blot. (B) The LACTB^M5L+R469K^ protein levels after Ixazomib treatment for 24h in SJSA-1 control group and LACTB^M5L+R469K^ knockdown group were detected by using western blot. (C) The wt-p53 protein levels after Bortezomib treatment for 24h in U2-OS control group and LACTB^M5L+R469K^ knockdown group were detected by using western blot. (D) The LACTB^M5L+R469K^ protein levels after Bortezomib treatment for 24h in U2-OS control group and LACTB^M5L+R469K^ knockdown group were detected by using western blot. (E) The wt-p53 protein levels after Ixazomib treatment for 24h in U2-OS control group and LACTB^M5L+R469K^ knockdown group were detected by using western blot. (F) The LACTB^M5L+R469K^ protein levels after Ixazomib treatment for 24h in U2-OS control group and LACTB^M5L+R469K^ knockdown group were detected by using western blot. (G) The LACTB^M5L+R469K^ protein levels after Leupeptin treatment for 24h in SJSA-1 control group and LACTB^M5L+R469K^ knockdown group were detected by using western blot. (H) The LACTB^M5L+R469K^ protein levels after Leupeptin treatment for 24h in U2-OS control group and LACTB^M5L+R469K^ knockdown group were detected by using western blot. (I) The wt-p53 protein levels after Leupeptin treatment for 24h in U2-OS control group and LACTB^M5L+R469K^ knockdown group were detected by using western blot. (J) The efficiency of siRNAs was detected by qPCR. (K) The changing of downstream genes of p53 pathway after PSMB7 knockdown were checked by qPCR. (L)(M) The protein levels of PSMB7 and wt-p53 after transfected with siRNA in SJSA-1 and U2-OS cells were detected by using western blot.


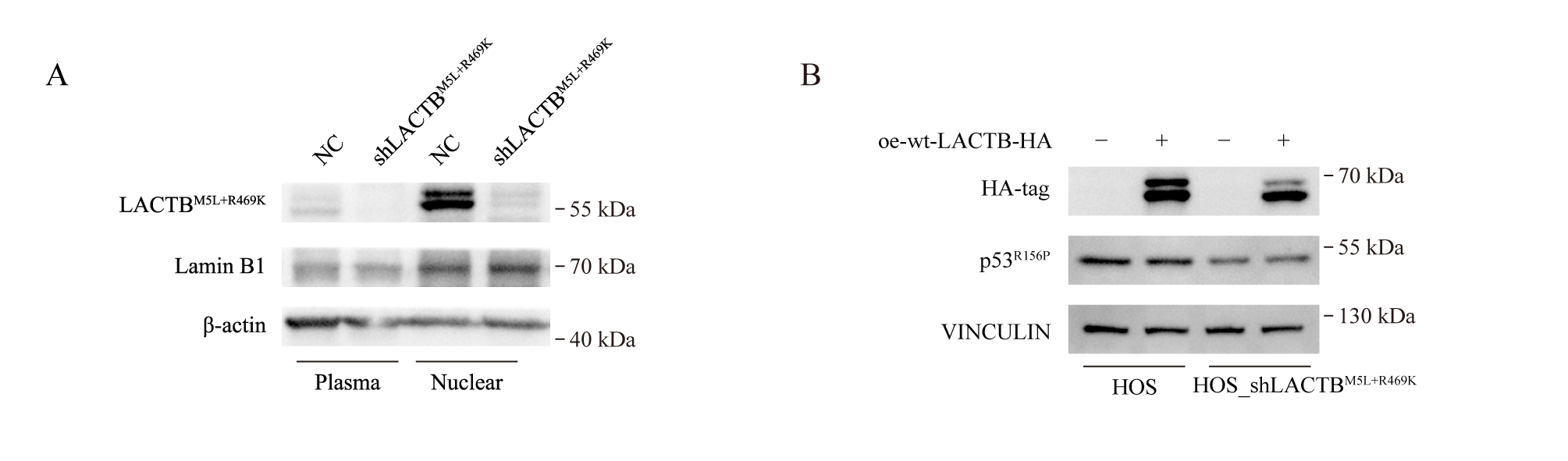


**Figure S7. The effect of wt-LACTB on p53^R156P^ in HOS cells.** (A) The LACTB^M5L+R469K^ protein levels in cytoplasm and nucleus were detected by using western blot. (B) The protein levels of p53^R156P^ after inducing wt-LACTB-HA overexpression in HOS control and LACTB^M5L+R469K^ knockdown cells were detected by using western blot.


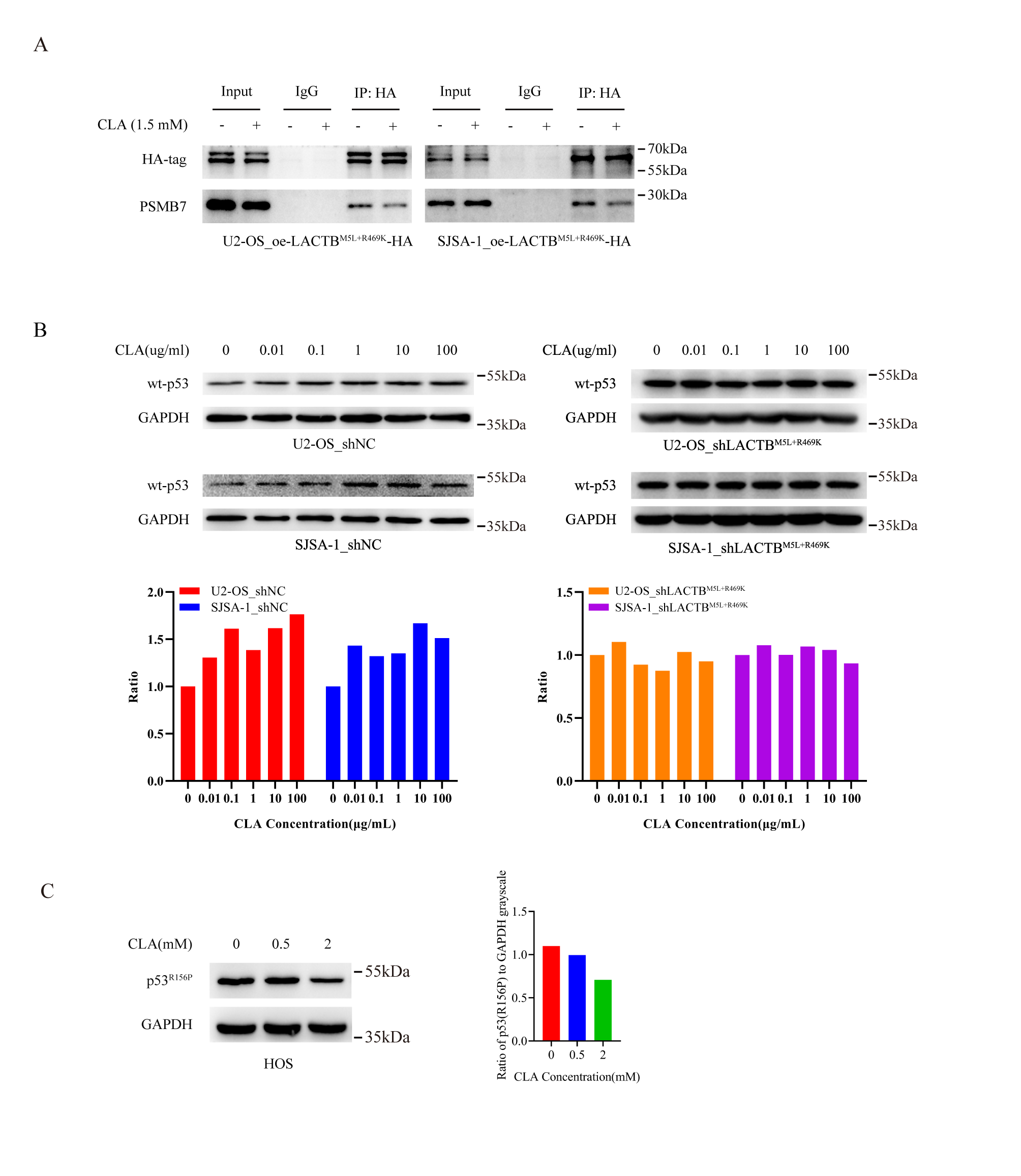


**Figure S8. Clavulanate potassium could disrupt the interaction between LACTB^M5L+R469K^ and PSMB7.** (A) The SJSA-1_oe-LACTB^M5L+R469K^-HA and U2-OS_oe-LACTB^M5L+R469K^-HA cells were treated with 1.5 mM CLA for 24h, and then applied co-IP by using HA-tag antibody. The protein levels of HA-tag and PSMB7 were detected by using western blot. (B) Different doses of CLA were used to induce wt-p53 protein expression in control group and LACTB^M5L+R469K^ knockdown group. The results were analyzed by western blot and grayscale analysis. (C) HOS cells were incubated with 0.5mM and 2mM CLA for 48h, and then the protein level of p53^R156P^ was checked by western blot and the grayscale analysis.

**
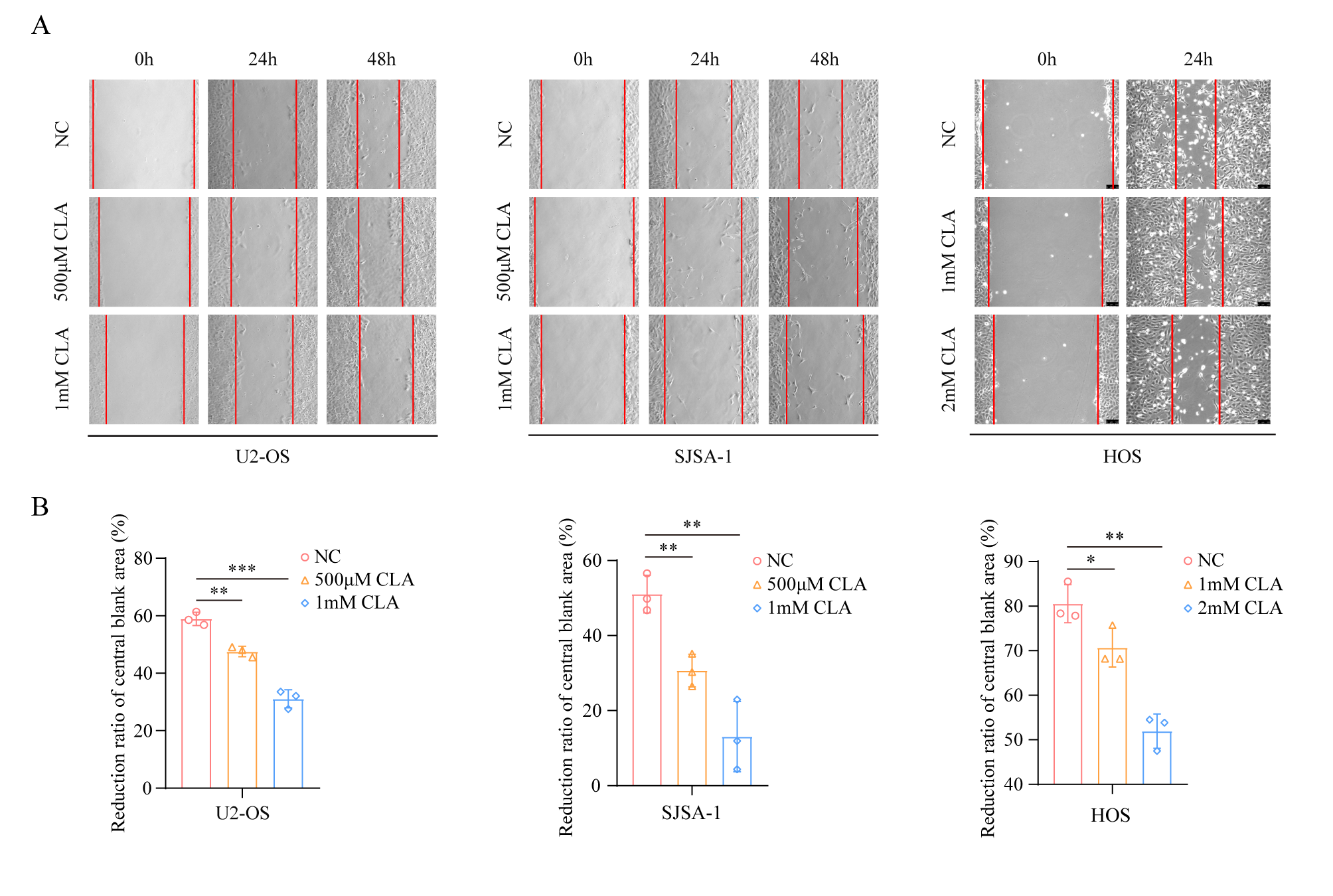
**

**Figure S9. Clavulanate potassium could inhibit migration of osteosarcoma cells.** (A) Scratch assays were used to evaluate the migration ability of U2-OS, SJSA-1 and HOS cells in control group and different dose CLA. (B) Calculating the percentage of reduction in the central blank area at the end of scratch assay in different group and conduct a statistical analysis.

**Table S1. The primers of TP53 pathway related genes.**

| Gene | Primer |
| --- | --- |
| TP53 | Forward Primer GAGGTTGGCTCTGACTGTACC  Reverse Primer TCCGTCCCAGTAGATTACCAC |
| BAX | Forward Primer CCCGAGAGGTCTTTTTCCGAG  Reverse Primer CCAGCCCATGATGGTTCTGAT |
| p21 | Forward Primer CGATGGAACTTCGACTTTGTCA  Reverse Primer GCACAAGGGTACAAGACAGTG |
| BAK1 | Forward Primer CATCAACCGACGCTATGACTC  Reverse Primer GTCAGGCCATGCTGGTAGAC |
| BMP2 | Forward Primer ACTACCAGAAACGAGTGGGAA  Reverse Primer GCATCTGTTCTCGGAAAACCT |
| BTG1 | Forward Primer AGCGGATTGGACTGAGCAG  Reverse Primer GGTGCTGTTTTGAGTGCTACC |
| BTG2 | Forward Primer ACCACTGGTTTCCCGAAAAG  Reverse Primer CTGGCTGAGTCCGATCTGG |
| CASP1 | Forward Primer TTTCCGCAAGGTTCGATTTTCA  Reverse Primer GGCATCTGCGCTCTACCATC |
| GADD45A | Forward Primer GAGAGCAGAAGACCGAAAGGA  Reverse Primer CACAACACCACGTTATCGGG |
| TAP1 | Forward Primer CGCCTCACTGACTGGATTCTA  Reverse Primer TCTGTTGGAAAAACTCCGTCTC |
| TXNIP | Forward Primer ATATGGGTGTGTAGACTACTGGG  Reverse Primer GACATCCACCAGATCCACTACT |
| PIGs | Forward Primer GCGGCTACACACCTAGAGG  Reverse Primer CTGGGAGTAAGGCAACGAGG |
| PIDD | Forward Primer GAGCCTCGTCGAGTCTCCAT  Reverse Primer GGCCCAGTACAACAGGTGC |
| PAI | Forward Primer GCACCACAGACGCGATCTT  Reverse Primer ACCTCTGAAAAGTCCACTTGC |
| P53R2 | Forward Primer AGAGGCTCGCTGTTTCTATGG  Reverse Primer GCAAGGCCCAATCTGCTTTTT |
| TSAP6 | Forward Primer CTCCCCGGAGGTCATCTTTG  Reverse Primer TCTTGCTCTGTAGGGTTGCTC |
